# Estimating the value of medical treatments to patients using probabilistic multi criteria decision analysis

**DOI:** 10.1186/s12911-015-0225-8

**Published:** 2015-12-02

**Authors:** Henk Broekhuizen, Catharina G. M. Groothuis-Oudshoorn, A. Brett Hauber, Jeroen P. Jansen, Maarten J. IJzerman

**Affiliations:** Department of Health Technology and Services Research, MIRA Institute, University of Twente, Enschede, The Netherlands; RTI Health Solutions, Research Triangle Park, NC USA; Department Public Health and Community Medicine, School of Medicine, TUFTS University, Boston, MA USA

**Keywords:** Multi criteria decision analysis, Patient preferences, Monte Carlo simulations, Probabilistic models, Depression

## Abstract

**Background:**

Estimating the value of medical treatments to patients is an essential part of healthcare decision making, but is mostly done implicitly and without consulting patients. Multi criteria decision analysis (MCDA) has been proposed for the valuation task, while stated preference studies are increasingly used to measure patient preferences. In this study we propose a methodology for using stated preferences to weigh clinical evidence in an MCDA model that includes uncertainty in both patient preferences and clinical evidence explicitly.

**Methods:**

A probabilistic MCDA model with an additive value function was developed and illustrated using a case on hypothetical treatments for depression. The patient-weighted values were approximated with Monte Carlo simulations and compared to expert-weighted results. Decision uncertainty was calculated as the probability of rank reversal for the first rank. Furthermore, scenario analyses were done to assess the relative impact of uncertainty in preferences and clinical evidence, and of assuming uniform preference distributions.

**Results:**

The patient-weighted values for drug A, drug B, drug C, and placebo were 0.51 (95 % CI: 0.48 to 0.54), 0.51 (95 % CI: 0.48 to 0.54), 0.54 (0.49 to 0.58), and 0.15 (95 % CI: 0.13 to 0.17), respectively. Drug C was the most preferred treatment and the rank reversal probability for first rank was 27 %. This probability decreased to 18 % when uncertainty in performances was not included and increased to 41 % when uncertainty in criterion weights was not included. With uniform preference distributions, the first rank reversal probability increased to 61 %. The expert-weighted values for drug A, drug B, drug C, and placebo were 0.67 (95 % CI: 0.65 to 0.68), 0.57 (95 % CI: 0.56 to 0.59), 0.67 (95 % CI: 0.61 to 0.71), and 0.19 (95 % CI: 0.17 to 0.21). The rank reversal probability for the first rank according to experts was 49 %.

**Conclusions:**

Preferences elicited from patients can be used to weigh clinical evidence in a probabilistic MCDA model. The resulting treatment values can be contrasted to results from experts, and the impact of uncertainty can be quantified using rank probabilities. Future research should focus on integrating the model with regulatory decision frameworks and on including other types of uncertainty.

## Background

Decisions in healthcare policy regarding research portfolio management, market access, reimbursement and price-setting all depend (in part) on the added value of medical treatments for patients. This treatment valuation task is difficult because it has to be based on a large set of (possibly uncertain) clinical evidence and on subjective assessments of the desirability of clinical endpoints. Multi criteria decision analysis (MCDA) is a decision analytic modelling approach that has been used for such treatment valuation tasks [1, 2], primarily because it can support decision makers by structuring the available evidence [3, 4] and by guiding informed discussions through visualizations [5]. In MCDA, the decision goal (in our case, valuing treatments) is decomposed into a set of concrete and measurable *criteria* (in our case, clinical endpoints or treatment characteristics like mode of administration). The identification of this set of criteria can be done, for example, by interviewing patients and clinical experts. Then, the set of relevant decision options (termed *alternatives*) is defined. These are often a given in a treatment valuation task. Now that the structure of the MCDA model is built, two main inputs are required: criterion weights and performance scores. Criterion weights indicate the relative importance of criteria. Performance scores measure the experts’ assessment of how well the alternatives perform on each of the criteria. Criterion weights and performance scores can be aggregated to come to an overall value of each included treatment [6]. This overall value can then be used to select a most preferred treatment, to rank treatments from best to worst, or to sort treatments into categories.

Studies applying MCDA to the treatment valuation task can, for example, be found in the decision contexts of market access [7–9] and reimbursement [10–12]. These applications of MCDA have mostly used expert input to construct the criterion weights and performances scores. However, it has been argued that the patient perspective forms an essential part of treatment value [13–16]. In an MCDA framework this could be operationalized by letting patients set the criterion weights. One approach for this is to involve individual patient representatives in the decision making process, but a more representative approach would be to use stated preference methods to elicit preferences from a large group of patients [17, 18]. These patient preferences could then be used to weigh the available clinical evidence [19]. In that way, treatment value can be estimated from the patient’s perspective in a transparent and representative manner. The results from such analyses could then be used as input for the decision makers’ decision making process.

In its simplest form, this combination of patient preferences with clinical evidence can be done deterministically. This would imply that the mean criterion weights and mean performance scores are used as input for the MCDA. However, including an assessment of uncertainty in a decision analysis would be advantageous because 1) it can help assess confidence in the outcomes of the model, 2) it can help ascertain the usefulness of performing additional research [20], and 3) can prevent bias in non-linear models [21]. Several approaches exist for taking into account uncertainty in MCDA. A recent review into uncertainty analysis approaches that are potentially useful for the specific context of healthcare identified deterministic sensitivity analysis, probabilistic sensitivity analysis, Bayesian frameworks, grey theory and fuzzy set theory [22]. The review concluded that deterministic sensitivity analysis is likely sufficient for most decisions in healthcare but that for decisions where the views of multiple stakeholders are combined or when uncertainty in multiple parameters is to be considered simultaneously, approaches that allow distributions (such as the probabilistic approach) would be more appropriate [22]. The treatment valuation task considered in this paper requires the combination of the views from multiple stakeholders (namely, a large group of patients) and requires the combination of uncertainty in multiple parameters (namely, all weights and performance scores), and the probabilistic approach is therefore adopted in this study.

The aim of this study is to illustrate how patients’ criterion weights derived from a stated preference study together with performance scores derived from clinical evidence can be used to value treatments from the patient’s perspective, taking into account parameter uncertainty in both criterion weights and performance scores. A hypothetical case based on earlier studies concerning three antidepressants and placebo will be presented to illustrate the developed model. Its main outputs are patient-weighted treatment values with associated 95 % confidence intervals. It will be shown how the patient valuation can be contrasted to an expert-based valuation and the utility of the developed modeling approach for practical decision making will be further illustrated by present the results from three scenario analyses.

## Methods

Suppose *I* treatments have to be valued in an MCDA based on *n* criteria simultaneously. We define treatments with a higher value to be preferred to treatments with a lower value. The clinical performance of drug *i* on criterion *k* is denoted with *θ*_*ik*_. The partial value function *v*_*k*_(*θ*) for criterion *k* maps the criterion-specific performance values *θ*_*ik*_ onto a linear scale between 0 at a ‘worst imaginable’ performance of *θ*_*k*_^−^ and 1 at a ‘best imaginable’ performance of *θ*_*k*_^+^ for treatment *i*:

1 $$ {v}_k\left({\theta}_{ik}\right)=\kern2.5em \left\{\begin{array}{cc}\hfill 1,\hfill & \hfill if{\theta}_{ik}\ge {\theta}_k^{+}\hfill \\ {}\hfill \frac{\theta_{ik}-{\theta}_k^{-}}{\theta_k^{+}-{\theta}_k^{-}},\hfill & \hfill \kern2.75em  if{\theta}_k^{-}<{\theta}_{ik}<{\theta}_k^{+}\hfill \\ {}\hfill \kern2.5em 0,\hfill & \hfill if{\theta}_{ik}\le {\theta}_k^{-}\hfill \end{array}\right. $$

The weights of the criteria are denoted with *w*_*k*_. These criterion weights indicate the relative importance of scale swings from *θ*_*k*_^−^ to *θ*_*k*_^+^ on a criterion, and should be estimated using the swing direct method or the MACBETH pairwise comparisons method [4]. To come to an overall value *V*_*i*_ for each treatment *i*, the partial values in this study are combined with an additive value function

2 $$ {V}_i={\displaystyle {\sum}_{k=1}^n{w}_k{v}_k\left({\theta}_{ik}\right),} $$

where it is assumed that the criteria are independent.

### Taking into account uncertainty

We adopt a probabilistic framework, in which the uncertainty in estimates for criterion weight and performance scores are represented with probability distributions [22]. The partial value functions and the overall values are stochastic variables with probability distributions that are complex combinations of the probability distributions for the weights and treatment performances. These are hard to calculate analytically and will therefore be approximated by applying a Monte Carlo simulation approach. In such an approach, for each simulation run *t*, weights *w*_*kt*_ and performances *θ*_*ikt*_ are sampled from their respective probability distributions. Then, formula’s 1 and 2 are used to come to partial values *v*_*k*_(*θ*_*ikt*_) and overall values *V*_*it*_. This process is then repeated a large number of times *T*.

The main outcomes of the MCDA model are the mean overall value for each treatment, the value distributions for each treatment, and the ranking probabilities for each treatment. The mean overall value for treatment *i* is estimated with the posterior mean, that is ${V}_i=\frac{{\displaystyle \sum }{V}_{it}}{T}$. The value distribution of treatment *i* is the empirical distribution of all *v*_*it*_. Rank probabilities are calculated by ranking treatments in descending order on their overall value each Monte Carlo simulation run. We define *r*_*xi*_ as the amount of Monte Carlo simulation runs were treatment *i* attains rank *x*. Then, treatment *i*’s rank probability for rank *x* is $\frac{r_{xi}}{T}$. The probability that the treatment with the highest mean value is not ranked first is used as a measure of decision uncertainty. It is calculated as follows: terming treatment *j* the treatment with the highest mean value, the probability that this treatment is not ranked first is $1-\frac{r_{1j}}{T}$.

### Illustration using a case

**Fig. 1:**
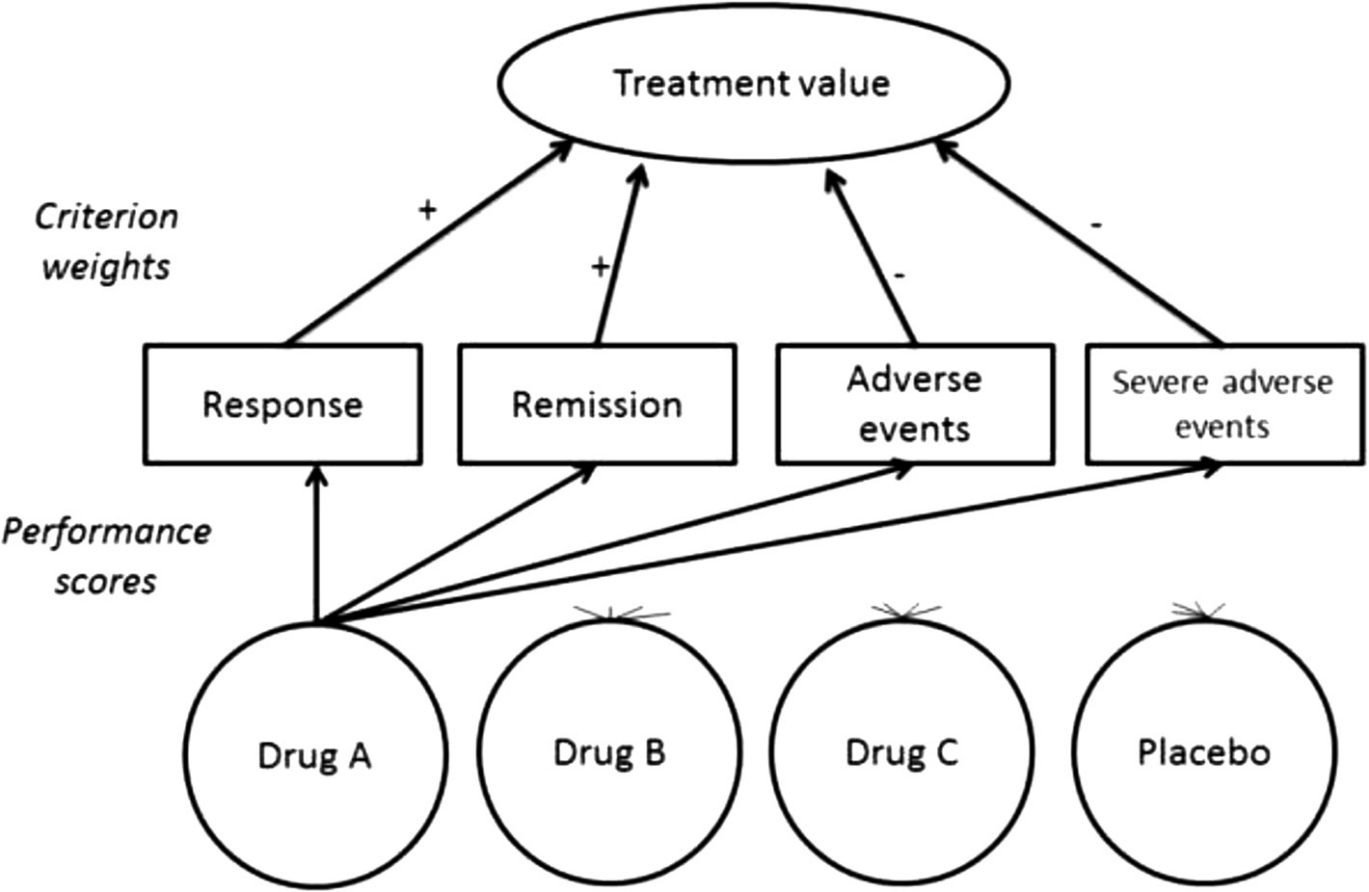
Decision structure used in the illustrative case. Starting from the top, there is the decision goal (assessing value), that can be operationalized with four criteria. The relative importance of the criteria is indicated by the criterion weights and the plus or minus indicates if the criterion is to be maximized or minimized. The performance of the four decision alternatives at the bottom on the criteria is determined with performance scores. Note that for clarity only the arrows showing the performance scores for drug A are shown

The model is illustrated with a case on treatments for severe depression. As can be seen in Fig. 1, the included treatments are compared on four criteria: response, remission, adverse events and severe adverse events. Response is defined as the probability of an acute 50 % reduction in depression symptoms as measured on a depression scale such as the Hamilton rating scale [23] for depression or the Montgomery Asberg depression rating scale [24]. Remission is defined as the yearly probability that depressive symptoms are reduced for such a time such that a patient can be considered to have recovered from an acute depressive episode. Adverse events considered are (yearly probability of) sexual dysfunction, hypertension, restlessness, sedation, dizziness, nausea, dry mouth, sweating and weight increase. Severe adverse events considered are (yearly probability of) suicide and other events that lead to death, threat to life, permanent/severe disability or hospitalization.

The response and remission criteria are to be maximized, with the best level *θ*^+^ defined as 100 % and the worst level *θ*^−^ as 0 %. The adverse events and severe adverse events are to be minimized, with the best level *θ*^+^ defined as 0 % and the worst level *θ*^−^ as 100 %. This means that, if for a patient the weight for response is 0.4 and the weight for remission is 0.2, that patient considers the swing from 0 % probability of response to 100 % probability of response to be twice as important as the swing from 0 % probability of remission to 100 % probability of remission when choosing an antidepressant.

For illustration purposes hypothetical preference and performance datasets were used. A patient criterion weight sample of 100 patients was constructed by bootstrapping the results from a patient panel held in an earlier elicitation study [25]. In that study, weights were also elicited from five clinical experts. These are included in our study for comparison with patient preferences. Three hypothetical antidepressants and placebo are included. We define drugs “A” and “B” as the currently used drugs. They are assumed to have moderate effectiveness and side effects. We assume that a large number of clinical trials have been performed over the years for drugs A and B and that therefore there is only minor uncertainty surrounding their clinical performance. We assume there is a new drug “C” that is potentially much more effective than the conventional drugs. However, we assume that due to its novelty only a small number of patients have been enrolled in clinical trials. This means there still is considerable uncertainty regarding its actual clinical performance. It is assumed that placebo provides almost no effectiveness and that it is associated with very little adverse events. It is assumed that all clinical trials ran for one year. An overview of the datasets for preferences and clinical performances is presented in Table 1.

**Table 1:** Hypothetical dataset used in the case study

*Criterion*	Direction	Patient weight (SD) (*n* = 100)	Expert weight (SD) (*n* = 5)	Drug A performance (events / n)	Drug B performance (events / n)	Drug C performance (events / n)	Placebo performance (events / n)
*Probability of response*	Maximize	0.46 (0.04)	0.01 (0.03)	5500 / 7000	6000 / 7000	84 / 100	250 / 1000
*Probability of remission*	Maximize	0.19 (0.02)	0.69 (0.07)	6000 / 7000	5000 / 7000	84 / 100	250 / 1000
*Probability of adverse event*	Minimize	0.14 (0.03)	0.13 (0.10)	300 / 7000	300 / 7000	1 / 100	5 / 1000
*Probability of severe adverse event*	Minimize	0.21 (0.02)	0.08 (0.08)	30 / 7000	30 / 7000	0 / 100	50 / 1000

Events = number of patients in trials that experience the event. *N* = total sample size of the trials

**Table 2:** Model outcomes: overall scores and rank probabilities

Scenario	Parameter	Drug A	Drug B	Drug C	Placebo
*Patients*	Score (95 % CI)	0.51 (0.48 to 0.54)	0.51 (0.48 to 0.54)	0.54 (0.49 to 0.58)	0.15 (0.13 to 0.17)
	P (Rank = 1)	10 %	17 %	73 %	0 %
	P (Rank = 2)	37 %	47 %	16 %	0 %
	P (Rank = 3)	53 %	36 %	11 %	0 %
	P (Rank = 4)	0 %	0 %	0 %	100 %
*Experts*	Score (95 % CI)	0.67 (0.65 to 0.68)	0.57 (0.56 to 0.59)	0.67 (0.61 to 0.71)	0.19 (0.17 to 0.21)
	P (Rank = 1)	51 %	0 %	49 %	0 %
	P (Rank = 2)	49 %	0 %	51 %	0 %
	P (Rank = 3)	0 %	100 %	0 %	0 %
	P (Rank = 4)	0 %	0 %	0 %	100 %
*Patients, only preference uncertainty*	Score (95 % CI)	0.52 (0.50 to 0.55)	0.53 (0.50 to 0.55)	0.55 (0.52 to 0.58)	0.15 (0.14 to 0.16)
	P (Rank = 1)	6 %	12 %	82 %	0 %
	P (Rank = 2)	36 %	49 %	14 %	0 %
	P (Rank = 3)	58 %	38 %	4 %	0 %
	P (Rank = 4)	0 %	0 %	0 %	100 %
*Patients, only performance uncertainty*	Score (95 % CI)	0.54 (0.51 to 0.56)	0.54 (0.51 to 0.57)	0.55 (0.50 to 0.60)	0.17 (0.16 to 0.19)
	P (Rank = 1)	15 %	26 %	59 %	0 %
	P (Rank = 2)	37 %	43 %	20 %	0 %
	P (Rank = 3)	48 %	31 %	20 %	0 %
	P (Rank = 4)	0 %	0 %	0 %	100 %
*Uniform distributions for criterion weights*	Score (95 % CI)	0.40 (0.12 to 0.68)	0.38 (0.11 to 0.66)	0.42 (0.14 to 0.70)	0.11 (0.02 to 0.20)
	P (Rank = 1)	33 %	28 %	39 %	0 %
	P (Rank = 2)	34 %	33 %	33 %	0 %
	P (Rank = 3)	31 %	37 %	26 %	7 %
	P (Rank = 4)	2 %	3 %	2 %	93 %

In each simulation run *t*, criterion weights *w*_kt_ are obtained by using a bootstrap resampling method. This means that for each run a bootstrap sample of 100 cases of the weight dataset was drawn with replacement. Because the clinical performances of drugs are proportions, performance samples *θ*_*ikt*_ are assumed to be distributed with a Beta distribution [21]. Beta distributions require two parameters: *α*_1_ and *α*_2_. We used the number of events as *α*_1_ and the sample size of the study minus the number of events as *α*_2_. This ensures that the expected value of the distribution is the event’s probability, and that the variance of the distribution is inversely related to the trial sample size. After sampling from the Beta distributions, *v*_*kt*_(*θ*_*ikt*_) and *V*_*it*_ are calculated using Formula’s 1 and 2. In total, *T =* 10,000 Monte Carlo simulations are performed. The 95 % confidence interval for each treatment’s value was estimated with the 2.5 %^th^ and 97.5 %^th^ quantiles of its *V*_*it*_ from the simulation output. The model was programmed in R [26].

Several scenario analyses will be performed. First of all, a model that uses only the mean criterion weights and mean performance scores will be run. Then, the impact of uncertainty will be explored by running separate Monte Carlo simulations with 1) only uncertainty in criterion weights (that is, fixing the performances at their mean values while varying the weights as in the base case), 2) only uncertainty in performance scores (that is, fixing the weights at their mean values while varying the performances as in the base case), or 3) uniform probability distributions for criterion weights (keeping the sum of weights constant at one, and varying the performance scores as in the base case).

## Results

### Patient and expert valuations of drugs

**Fig. 2:**
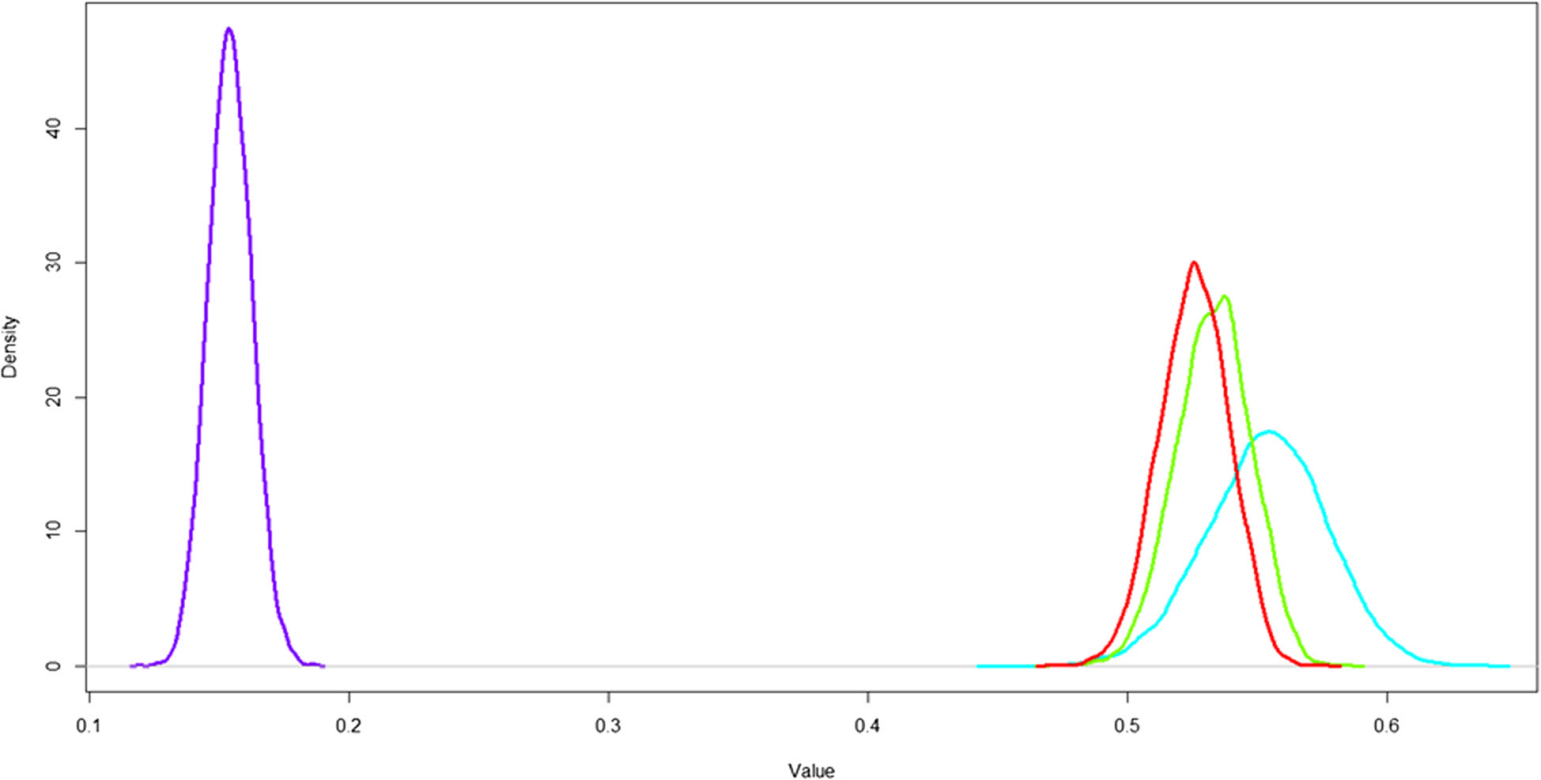
Probability density estimation plot (Gaussian kernel estimation using the *density* function in R) of the model results for when patient preferences are used. Red = Drug A, green = Drug B, Blue = drug C and purple = placebo. Treatment value distributions in base case

When using a deterministic model, that is, setting both criterion weights and performance scores to their mean values, the overall scores for drug A, drug B, drug C and placebo are 0.51, 0.51, 0.54, and 0.15, respectively for patients. For experts, the overall scores for drug A, drug B, drug C and placebo would be 0.67, 0.57, 0.67, and 0.19, respectively. This suggests that drug C has the highest value for patients and drugs A and C seem the most valuable treatment according to experts. Although this is already an insightful result, we cannot assess the confidence of these valuation statements. Taking into account uncertainty as described in the Methods section gives us more insight into the treatment valuation by patients and experts (Fig. 2). Note the more spread out probability density for the value of drug C which indicates that its value is more uncertain than that of drugs A and B. Drug C seems still to be the most valuable treatment to patients with a probability of being ranked first of 73 %. Placebo still has the last rank, as would be expected. There is considerable uncertainty in the treatment values: there is a 27 % probability that drug C turns out to not be the most valuable to patients. Furthermore, there is considerable decision uncertainty as to the second most valuable drug (*r*_2__*A*_ = 37 % (and *r*_2__*B*_ = 47 %). The clinical experts’ results incorporating uncertainty show that drugs A and C that both have a score of 0.67. The first rank probabilities for drug A and drug C are 51 % and 49 %, respectively. This means there is clinical equipoise between drugs A and C according to experts. Drug B is ranked third in all simulations with a score of 0.57 and placebo is again ranked last in all simulations with a score of 0.19. The impact of patient preferences as opposed to clinical experts is thus that while patients seem certain that drug C has the highest value, experts consider drugs A and C to be equally valuable. An overview of drug values and rank probabilities can be found in Table 2.

**Fig. 3:**
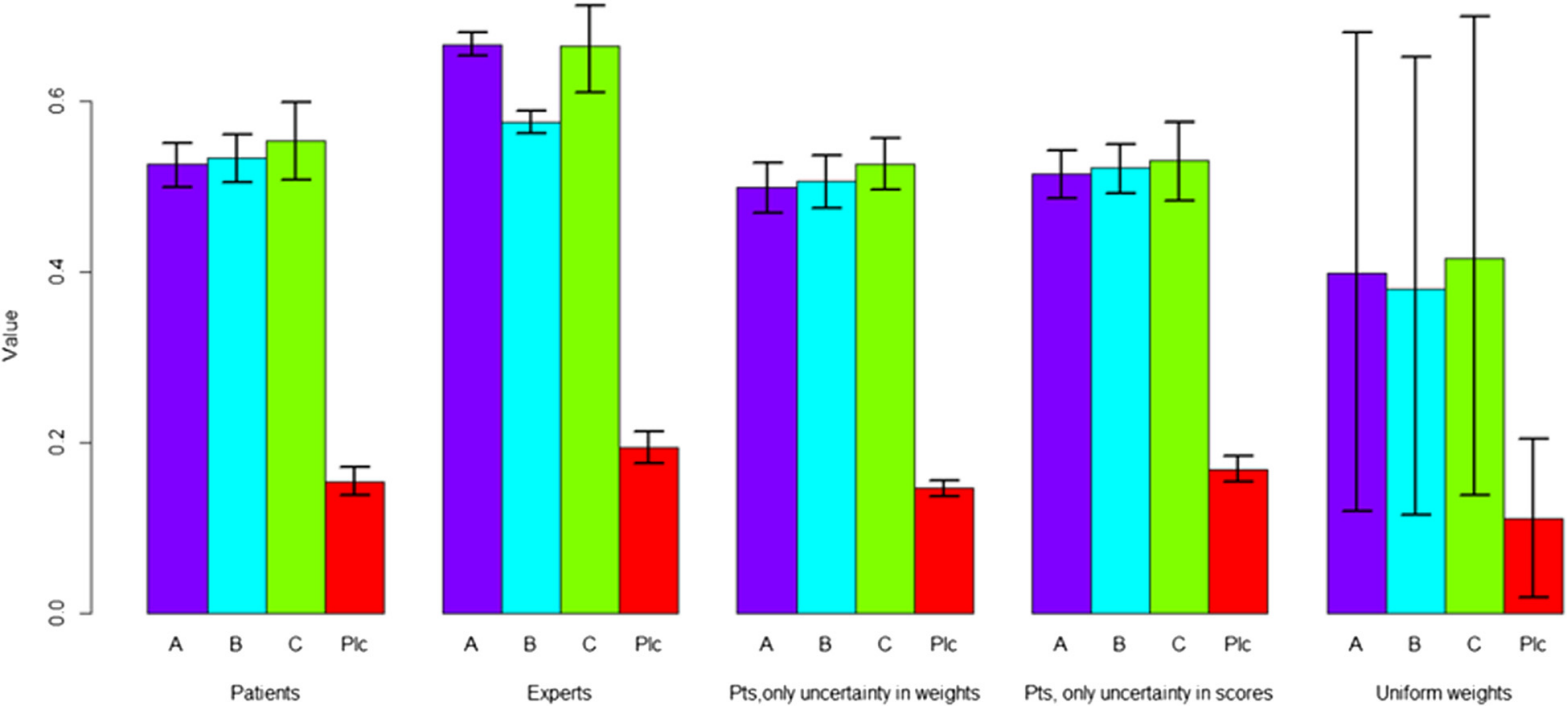
Overview of the overall values of the included treatments. From left to right: patients (with uncertainty in weights and performance scores), experts (with uncertainty in performance scores), patients (with uncertainty in weights but no uncertainty in performance scores), patients (with uncertainty in performance scores but no uncertainty in weights), uniformly distributed weights (with uncertainty in performance). The error bars indicate the 95 % confidence intervals. Pts = Patients, Plc = Placebo

### Impact of uncertainty

The results from the scenario analyses as compared to the base case are presented in Figure 3. When uncertainty in either patient-assigned criterion weights or performance scores is ignored (that is, set to their mean values), the point estimates for all four drugs remain similar. However, the confidence intervals of the drugs become smaller. This can be seen also in the ranking probabilities, which are higher for each rank. The rank reversal probabilities for first rank decreases to 18 % when uncertainty in performances is not taken into account and increases to 41 % when uncertainty in criterion weights is not taken into account. This means that in the case performance uncertainty seems to have a larger impact than preference uncertainty on the confidence with which the most valuable drug is chosen. Finally, using a uniform distribution for criterion weights induced a very large variation in drug scores and consequently, a high rank reversal probability for the first rank (61 %). This large variation in scores is logical because the criterion weights vary between 0 and 1, whereas in the other scenarios there is much less variation in sampled weights (Table 1).

## Discussion

In this paper we have demonstrated a probabilistic multi-criteria approach to determine the patient-weighted value of treatments. The MCDA model developed for this purpose takes into account the parameter uncertainty surrounding both the elicited preferences and clinical trial data. The model was illustrated using a hypothetical case on three antidepressant treatments and placebo. In the case the patient-weighted treatment values are considerably different from the expert-weighted values. Furthermore, the rank order of treatments is still uncertain for patients and experts (as reflected in the rank reversal probabilities). Scenario analyses showed that in this case decision uncertainty seems to depend more on uncertainty in clinical evidence than on uncertainty in patient preferences. Finally, adopting uniform criterion weight distributions lead to the most decision uncertainty, as reflected in the high probabilities of rank reversal.

### Comparison to earlier work

Our MCDA model builds on and combines characteristics from earlier approaches for evidence gathering and evidence synthesis. First of all, the model structure and value functions are based on value-based MCDA [3, 7]. There various “families” of MCDA methods, each with their own (dis) advantages. In this paper a value-based method based on multi-attribute value theory was used. The main advantages of this method are its strong foundation in decision theory [27] and the ease of weight elicitation (which is especially relevant when patient preferences are used). Secondly, preference data from stated preference methods can be included in the model, allowing the incorporation of uncertainty around patient preferences. Thirdly, this uncertainty was combined with uncertainty around clinical performance estimates using Monte Carlo simulation methods. Although there have been other methods to combine patient preferences and clinical trial data in the context of healthcare policy, these are mainly limited by not practically taking into account multiple (concurrent) events and/or uncertainty around preferences [28–32]. Stochastic multi-criteria acceptability analysis (SMAA) also combines preference data with clinical trial data, but a non-informative (uniform) distribution or a single rank order of criteria is used for preferences [19, 33]. A similar approach is adopted by Caster et al*.* who include a rank order of criteria importances based on qualitative information on utilities [34]. Although both SMAA and the method by Caster et al*.* can include information about patient preferences, only including rank orders of criteria would preclude decision makers from considering the rich information on patient preferences yielded by stated preference studies.

### Applicability and advantages of the model

The treatment valuation task considered in this paper forms only one ingredient of healthcare policy decisions. This is because there is a distinction between a patient’s preferences and values, the patient’s health-related behavior, and the actual implementation of a decision in the context of a specific healthcare system. Although these concepts are clearly linked, the main distinction is that behavior and outcomes *may or may not* be in line with a patient’s preferences, depending on constraints concerning the patient’s circumstances, his/her behavior, and/or the context of the specific healthcare system. After establishing the value of treatments to patients using our model, further modelling work, e.g. with dynamic (system) simulation models [35], or fuzzy cognitive maps to estimate patients’ behaviors [36], may support decision makers design policies that are best in line with the patient’s preferences. On a physician-patient interaction level where (for example) prescription decisions are made, decision aids (based on MCDA for example [37]) that help patients think about their preferences and the treatment options may be valuable, but the probabilistic modelling framework adopted in this study may be prohibitive with regard to time constraints.

Although this was a demonstration and not an empirical comparison of the model to other modes of decision making, we believe the presented approach may have several advantages for decision makers seeking to do a treatment valuation task as part of their decision making process. First of all, the adopted MCDA approach can help decision makers to structure the available preference data and clinical evidence and can help them assess the impact of preferences on the overall value of treatments. The present study adds to this the explicit inclusion and combination of patient preferences and clinical evidence. Furthermore, to account for uncertainty in both preferences and clinical data, the flexible probabilistic approach is adopted. These two additions may give decision makers more insight into 1) the influence of patient preferences on treatment value, and 2) into the impact of uncertainty in both preferences and clinical data on the decision. A final advantage is that because of the explicit use of evidence and the use of visualizations, decision makers can use the model to communicate their decision (argumentation) to stakeholders. This can be especially true for communicating a decision to patients because patient preferences are explicitly used.

Our model considers mainly the evidence-based treatment valuation task, whereas a complete regulatory decision making process in healthcare has much more steps. Therefore, real-world applications of our model would require it to be applied in the context of an overarching decision making framework that guides the decision making process from problem definition to final decision. One such framework is PrOACT-URL, which structures the decision making process with the following phases: problem, objective, alternatives, consequences, trade-off, uncertainty, risk tolerance and linked decisions [38]. For an inclusion of the developed model in PrOACT-URL, criterion weights should be elicited from patients in the process after the definition of the ‘effects table’ in the consequences phase. These could be combined with the clinical evidence according to the model developed in this study to help decision makers assess the benefit-risk balance from a patient’s perspective in the trade-offs phase of PrOACT-URL and to guide the discussion in the uncertainty phase that follows the ‘trade-offs’ phase. We argue that the inclusion of our proposed model into frameworks such as PrOACT-URL would be most useful when it is judged by decision makers that the decision to make is characterized by uncertain clinical evidence and/or uncertain patient preferences. Given the explicit use of elicited patient preference, decision makers seeking to apply our model should be aware of remaining normative issues regarding the use of elicited patient preferences in real world decision contexts. These are: whose preferences should be elicited, who should perform the preference elicitation study, and what stated preference method should be used.

Aspects of the decision making process that may change in real world policy decision contexts compared to our simple illustrative case, are that more patients could be involved and that more criteria (not all relating directly to the patient experience) may be considered relevant by the decision makers. Given previous experience with performing large patient preference studies [17, 18, 39] and experience with using MCDA to consider large amounts of criteria [3, 40], it is reasonable to expect that our model can be extended to real world use. Furthermore, even if the real-life decision involves other criteria requiring other normative judgments outside the patient experience (such as societal willingness to pay), it is possible to construct an MCDA model that includes the preferences of multiple stakeholder groups. The relative weight of the preferences of these (and potentially other) stakeholder groups can then be weighed by the decision makers, who make the final decision after considering the outcomes of the evidence synthesis as facilitated by the MCDA model presented in this study. Finally, in the case of benefit-risk assessments decision makers may be reluctant to aggregate benefits and risks into one score [29]. In that case, decision makers could elect to model benefits and risks in separate MCDA models and use the results during the assessment of the benefit-risk assessment.

### Limitations of the model and opportunities for further research

Our model has some limitations. First of all, a multi-attribute method with a simple additive value function as aggregation method was used in this study. Although more complex methods are known, adopting such an aggregation method may imply that the elicitation questions become too hard for patients to understand. Independency of criteria is assumed in our MCDA model. In real-world applications of the model this requires great care to be taken when the decision model is built together with the stakeholders since it is essential that the included criteria comply with the assumptions in the MCDA model. Future studies could use modeling strategies for example with joint distributions of preference parameters such that the independency assumption can be relaxed. Another limitation in this study was that the overall treatment value was assumed to scale linearly with the criterion weights. Since the lower and upper levels were 0 % and 100 % for all criteria in the case, this implied that criterion weights reflected the relative importance of events and that (often reported) non-linear preferences for probabilities could not be incorporated, although methods are known for eliciting non-linear value functions from respondents (e.g. the bisection method [27]).

There are several categories of uncertainty in MCDA [22]. In this study, only parameter uncertainty was considered, while patient-specific preference variation and patient-specific variation in outcomes is increasingly becoming important in light of recent developments in personalized medicine [41]. A final and practical limitation is that the process of gathering relevant data on patient preference and clinical evidence, as well as building the model can be time-consuming. What MCDA methods to use in real-world applications of the presented model should be the topic of future research. Aspects we believe are important include the type of patient preferences that are to be elicited (since these need to match the MCDA method [42]), the preferable type of clinical evidence and specific decision maker needs. It may be useful to look into experiences in other disciplines where there is a longer history of using MCDA to support decision makers (see e.g. [43–46]).

## Conclusions

In conclusion, we have developed a novel approach to estimate the value of treatments from the patient perspective using a probabilistic MCDA model. The model was illustrated with a case on antidepressants. The model can provide insight into the patient-weighted value of treatments and how this may differ from an expert’s assessment. It also can provide insight into the impact of uncertainty that still surrounds the value of treatments. Future work will need to address patient-specific variation and the feasibility of the modeling approach in practical applications (specifically in existing regulatory decision making frameworks).

## Abbreviations

CI: confidence interval
MACBETH: measuring attractiveness by a categorical based evaluation technique
MCDA: Multi criteria decision analysis
PrOACT-URL: problem, objective, alternatives, consequences, trade-off, uncertainty, risk tolerance and linked decisions
SMAA: stochastic multicriteria acceptability analysis
